# Comparative Analysis of Cognitive and Psychiatric Functioning in People With Cushing's Disease in Biochemical Remission and People With Nonfunctioning Adenomas

**DOI:** 10.1155/2024/4393169

**Published:** 2024-11-13

**Authors:** Mary A. Fernandes, Sabrina D. Hickle, Suzanne Penna, Adriana G. Ioachimescu, Erin B. Tone

**Affiliations:** ^1^Washington D.C. Veterans Affairs Medical Center, Washington, DC 20422, USA; ^2^Department of Rehabilitation Medicine, Emory University, Atlanta, Georgia 30322, USA; ^3^Department of Medicine (Division of Endocrinology and Molecular Medicine) and Neurosurgery, Medical College of Wisconsin, Milwaukee, Wisconsin 53226, USA; ^4^Department of Psychology, Georgia State University, Atlanta, Georgia 30303, USA

**Keywords:** cognitive functioning, Cushing's disease, hypercortisolism, nonfunctioning pituitary adenoma, psychological functioning, remission

## Abstract

People with Cushing's disease (CD) often experience both mood/anxiety disorders and cognitive impairments that persist during long-term biochemical remission. The relationship between persistent neurocognitive and psychiatric problems in patients with CD is not well understood. Also, mechanisms other than hypercortisolism are poorly understood, and studies comparing CD with nonfunctioning adenomas (NFA) patients postoperatively are scarce. We compared neuropsychological functioning in two groups: individuals with CD in remission (*n* = 20; 80% female; 61.6 [44.13] months since remission) and individuals with NFAs (*n* = 20). Evaluation was performed, on average, 4.9 years following pituitary surgery. We used mediation models to evaluate psychiatric dysfunction as a possible mediator of cognitive outcomes and assessed the influence of demographic and medical factors (age at diagnosis, remission duration, and radiation therapy) on neuropsychological outcomes. Neuropsychological outcomes did not differ significantly between groups; however, up to 30% of patients demonstrated mild impairments in attention, processing speed, executive functioning, and visual memory. Time since remission in the CD group was inversely correlated with processing speed; however, this relationship was no longer significant after controlling for the presence of hypertension and diabetes mellitus. Levels of anxiety, depression, or somatization were reported in up to 40% of people with CD. Further, 70% of people with CD and 35% of people with NFA reported continuous depressive symptoms lasting at least 2 years. In conclusion, neuropsychological screening in clinical practice and longitudinal studies in individuals with NFA and CD are needed to identify patients at risk for long-term neuropsychological dysfunction. Appropriate support and treatment are recommended for persistent cognitive and/or psychiatric dysfunction for both patient groups.

## 1. Introduction

Endogenous Cushing's disease (CD) is an endocrine system disorder caused by chronic overproduction of cortisol in the context of an adrenocorticotropic hormone–producing pituitary adenoma. Primary treatment is transsphenoidal surgery (TSS), with radiation and/or medications used in patients who do not achieve remission with TSS alone [1]. Research has linked chronic exposure to excess cortisol with changes in brain structure and function, as well as cognitive and psychological dysfunction [2]. Perhaps unsurprisingly, then, in its active phase, CD is often associated with psychiatric symptoms such as depression (80%–90%) [3, 4], anxiety (66%) [5], and mania (30%) [6], as well as neurocognitive impairments in memory and concentration [7, 8]. These impairments often persist, sometimes for years [2, 7], despite biochemical remission, defined as the resolution of hypercortisolism following surgery.

Given the high concentration of glucocorticoid receptors in the hippocampal region, most research on postremission cognitive function has focused on memory, showing postoperative impairments in verbal and nonverbal memory [9]. Only a handful of studies have evaluated other cognitive domains, such as processing speed, visual–spatial skills, or attention/working memory [10, 11]. Even fewer studies have assessed long-term psychiatric outcomes for individuals with CD [7, 12]. A more comprehensive cognitive and psychiatric profile among people with CD in remission is needed. Research to date also leaves unclear the degree to which atypical cognitive and psychological functioning among individuals with CD in remission results from chronic hypercortisolism and/or from other factors related to tumor mass effects (e.g., hypogonadism, hyperprolactinemia, and hypothalamic dysfunction) [13]. A direct comparison of functioning between remitted people with CD and those with nonfunctioning adenomas (NFAs, adenomas of the pituitary gland without associated excessive hormone production) would help isolate outcomes that are specifically associated with chronic hypercortisolism. Of the few studies that included an NFA control group, findings indicated poorer cognitive functioning and increased prevalence of psychopathology among individuals with CD [11, 12]; however, these studies did not assess cognitive and psychiatric functioning in conjunction.

Additionally, although individuals with CD in remission exhibit both cognitive and psychiatric problems [2, 14], it is not known whether or how they may operate in tandem. In particular, in light of evidence that anxiety and depression are associated with cognitive impairments, it is possible that psychological dysfunction plays a role in maintaining cognitive impairments among individuals with remitted CD.

Finally, in exploratory analyses, we examined associations among demographic and health factors (age at diagnosis, hypogonadism, time since remission, and duration of hypercortisolemia) and cognitive and psychological outcomes among people in remission from CD. Given that these factors have been identified as either conferring increased risk for or protecting against relapse and/or mortality, it was important to assess how they relate to neuropsychological functioning in the present remitted sample.

To summarize, in the present study, given evidence of diffuse white matter deterioration [15] and functional connectivity abnormalities in the medial temporal lobe and prefrontal cortex [16], we first tested the hypothesis that individuals with CD would display poorer performance on tasks measuring attention, executive functioning, visual–spatial functioning, memory, and processing speed than those with NFAs. Further, given the well-documented adverse effects of cortisol on mood and anxiety, as well as evidence of prolonged cortisol effects on areas of the brain involved in affect regulation [16], we expected people with CD to endorse higher levels of negative affect, anxiety, and somatization and lower positive affect than people with NFA. Second, we assessed the contribution of psychiatric functioning to cognitive outcomes in a partial mediation model. Finally, we conducted an exploratory assessment of associations between other factors on neuropsychological outcomes among individuals in remission from CD. These included age at diagnosis, duration of hypercortisolism, duration of remission, and presence of hypogonadism, which may impact neuropsychological outcomes after remission.

## 2. Methods

### 2.1. Recruitment

Potential participants with CD were recruited among patients treated through the Emory Pituitary Center, a multidisciplinary clinical service that offers specialty medical care for patients with pituitary tumors and other pituitary conditions. Potential participants were identified based on their diagnosis and review of their medical charts supportive of biochemical remission, defined as low or normal serum cortisol achieved as a result of surgery and/or postoperative radiation of the pituitary tumor. A research team member phoned individuals who appeared to meet study criteria, described the study and answered questions, and conducted a brief screen for fit with inclusion criteria (age between 18 and 75 years; clinical and biochemical diagnosis of CD; up-to-date endocrine testing in order to confirm remission status; no history of head injury, major neurological disorder, recent substance use disorder, or other psychological, medical, or learning conditions that would preclude completing a 120-min test battery). Interested individuals provided informed consent via encrypted email and were then assigned a study ID and sent web links to computerized neuropsychological tests and surveys measuring psychological symptoms. Hormone measures, as well as treatment (transsphenoidal surgical tumor removal or radiation), were obtained from participants' medical records. NFA controls were matched to CD participants based on age (within 10 years), race, and gender. All individuals with NFA underwent surgery within a similar timeframe as the group with CD. Individuals with CD who could not be matched with same-race individuals with NFA, due to the rarity of the disease, were matched with people with NFA from other, non-White racial groups. Where possible, controls were also matched based on treatment history, resulting in 18 CD-NFA pairs that were accurately matched based on treatment history (TSS and/or radiation).

Power analyses indicated that with a total sample size of 34 or larger, we were adequately powered (1‐*β* = 0.80) at the 0.025 alpha level to detect a medium-sized effect (*f* = 0.25) [17] of two groups (CD vs. NFA) on cognitive and psychological functioning (six variables) using a multivariate analysis of variance (MANOVA) [18] and also adequately powered (1‐*β* = 0.59) at the 0.05 alpha level to detect a large mediation effect (*β* = 0.59) [19] using bias-corrected bootstrapping.

**Figure 1:**
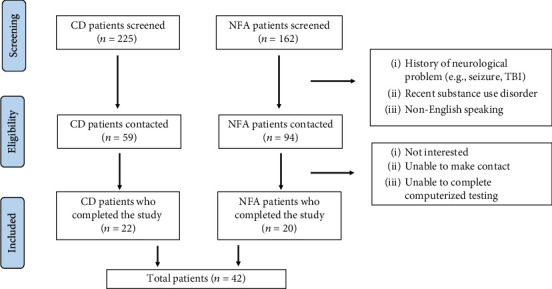
Flow chart of participant identification and inclusion.

In order to achieve our intended sample size, we screened 225 people with CD and 162 people with NFA. As shown in Figure 1, 59 people with CD patients and 94 NFA patients met the study criteria and were contacted regarding participation. Of the 37 individuals with CD who were successfully reached, 22 completed the study (59% completion rate), and of the 41 eligible candidates with NFAs with whom contact was made, 20 completed the study (49% completion rate). The final sample thus comprised 42 individuals (22 CDs and 20 NFAs), with 20 matched pairs (*n* = 40) that were included in analyses.

See Table 1 for demographic, medical, and treatment characteristics of the sample. Participants underwent surgery between 2002 and 2019 by the same neurosurgeon. Compared to a broader sample of CD patients who underwent TSS in the Metropolitan Atlanta area at the same institution [20], more White individuals participated in this study, while Black individuals were underrepresented.

This study's CD sample is comparable to other CD samples with respect to average age at diagnosis (39.5 years) [21] and average estimated time from symptom onset to diagnosis (27.7 months) [21]. Hypertension and diabetes mellitus (DM) were most often endorsed as persistent comorbidities, consistent with previous studies [22].

### 2.2. Measures

#### 2.2.1. Cognitive Measures

To ensure safety during the COVID-19 pandemic, all testing was conducted remotely via computer using online-accessible neuropsychological tests and questionnaires. Neuropsychological measures were drawn from the TestMyBrain (TMB) Digital Neuropsychology Toolkit, which is funded by the US National Institutes of Health and was disseminated for free use during the COVID-19 crisis. Normative data for the TMB battery, collected from samples of 4000–60,000 individuals aged 12–90 years, are stratified by age, gender, level of education, and type of device used. Subtests included the *Digit Span* test (a measure of simple auditory attention/working memory), the *Gradual Onset Continuous Performance Test* (a measure of sustained attention/vigilance), *Digit Symbol Matching* (a measure of processing speed), *Matrix Reasoning* (a measure of perceptual abstract reasoning), *Visual Paired Associates Memory* (visual learning/memory), *Verbal Paired Associates Memory* (verbal learning/memory), *Trail-Making Test Parts A and B* (a measure of processing speed and executive functioning), and *Choice Reaction Time (*processing speed and executive functioning). For more information about these measures and the domains that they measure, please refer to the Appendix.

TMB tests (Coding, Matrix Reasoning, and Digit Span) that were developed based on the Wechsler Adult Intelligence Test-IV (WAIS-IV) [23] show acceptable correlations with traditional WAIS-IV tests [24]. The remaining tests, including the TMB Gradual Onset Continuous Performance Test, Trail-Making Test Parts A and B, Choice Reaction Time Test, and Verbal and Visual Paired Associates Memory Tests, similarly show parallel psychometric characteristics with in-person, lab-based versions [25]. Three well-validated psychological functioning measures of anxiety (TMB GAD-7) [26], depression (TMB PHQ-8) [27], and general functioning related to a health condition (TMB WHODAS 2.0) [28] were also administered via the online toolkit.

#### 2.2.2. Emotional/Psychiatric Measures

In addition to the toolkit, participants completed a Qualtrics survey consisting of several psychological measures. The Positive and Negative Affect Schedule (PANAS) Short Form [29] is a 20-item scale that assesses positive and negative emotions experienced over the past week. The Brief Fear of Negative Evaluation Scale—Straightforward Version [30] is an 8-item measure that assesses fear about others' evaluations, distress over negative evaluations, and expectations of negative evaluations. The PHQ Somatic Symptom Scale (PHQ-15) [31] is a 15-item scale that measures commonly experienced somatic complaints.

#### 2.2.3. Psychosocial Stressors

Exposure to various difficult or stressful life events (e.g., natural disasters, combat, and accidents) was measured by the Life Events Checklist [32]. In addition to completing validated, self-reported measures of psychiatric functioning, participants also reported their history of major and/or persistent depressive disorder. Given that weight gain is 95%–100% prevalent among CD patients, the Stigmatizing Situations Inventory, adapted from Myers and Rosen [33], was also administered, which assesses weight-related perceived stigma in healthcare settings. Finally, to capture COVID-related psychological and physical health concerns, the COVID Stress Scale (CSS) [34] and the Coronavirus Stressor Survey, adapted from McLean and Cloitre [35], were included in the battery.

### 2.3. Data Analysis

Participant characteristics (gender, race, ethnicity, age, and years of education) are presented for the overall sample and for each group (CD and NFA; see Table 1). Independent two-sample *t*-tests and chi-square analyses were conducted to compare demographic variables (age, gender, race, and years of education) between the CD and NFA groups.

We then conducted two MANOVAs, one assessing six cognitive domains (attention/working memory, processing speed, visuospatial skills, verbal memory, visual memory, and executive functioning), and the other assessing six psychiatric domains (anxiety, depression, health and disability, somatic complaints, social anxiety, and positive emotions), with diagnosis as the grouping variable.

Post hoc independent two-sample *t*-tests were conducted to compare age-normed *z*-scores from each of the cognitive tests included in MANOVAs between groups. For cognitive domains that included multiple tests measuring the same construct (e.g., attention measured by TMB Forward Digit Span and Gradual Onset CPT Detectability and Impulsivity), *z*-score composites of the scores from relevant measures for each domain were included in the *t*-tests. Total scores from each of the psychological measures were also included as independent variables in *t*-tests.

In addition to simple comparisons between the two groups, in a third set of analyses, *z*-score cutoffs were applied to identify individuals who performed poorly at an impaired level. We set the cutoff to 1 standard deviation below the normative mean in order to capture “mild” impairments that might be more prevalent in this patient population [36]. Chi-square tests were conducted to test whether the number of participants identified as impaired or intact when applying a cut-off of *z*‐score ≤ −1 differed between the two groups. The same impairment/intact categorization was applied to the psychological measures using the cutoff scores for each measure. Given that the WHODAS 2.0 was designed as a descriptive rather than classifying measure, cutoff scores are not available. Therefore, the WHODAS 2.0 was excluded from this third set of analyses only.

We conducted three mediation analyses, each with a different outcome variable (attention, memory, and executive function *z*-score composites calculated for attention and executive functioning in Aim 1 analyses). For the mediation model with memory as the outcome variable, we created a *z*-score composite of the TMB Visual and Verbal Paired Associates Memory Tests as the memory outcome variable. A *z*-score composite of the TMB PHQ-8 and the TMB GAD-7 was created and used as the moderating variable. Group membership (CD vs. NFA) served as the independent variable. To test this mediation model, we used the PROCESS macro for SPSS [37] resampling 10,000 times.

Associations between other factors (age at diagnosis, duration of hypercortisolism, presence of hypogonadism, and duration of remission) and cognitive and psychological functioning were examined in exploratory analyses. The duration of hypercortisolism was estimated by a retrospective review of participants' medical charts and the participants' self-report of duration of symptoms prior to diagnosis. Time since remission, cortisol status, and presence or absence of hypogonadism were determined by a retrospective review of the participants' medical charts.

We conducted bivariate correlations between each of the cognitive and psychological domains and age at diagnosis, duration of hypercortisolism (estimated by the number of months since the first symptom was noticed until remission was achieved), and time since remission. We also conducted chi-square tests to determine if cortisol status (hypocortisolemia or normal cortisol production) and the presence or absence of hypogonadism were associated with cognitive and psychological functioning.

## 3. Results

CD and NFA samples did not differ significantly on any demographic variable (Table 1). People with CD had a higher prevalence of hypertension and DM than people with NFA. People with CD achieved biochemical remission as a result of surgery alone (*N* = 17) or surgery and postoperative radiation (*N* = 3).

Given that all MANOVA assumptions were met, we proceeded by conducting two MANOVAs to detect group differences in cognitive and psychological functioning. Neither model yielded significant results.

**Figure 2:**
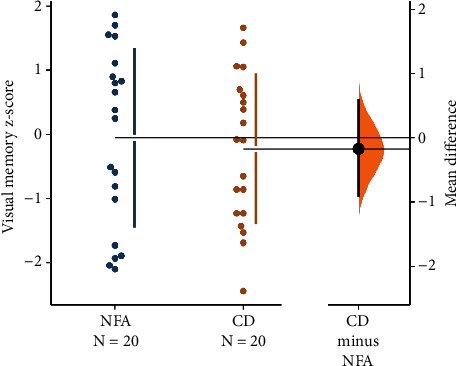
Two-group estimation plot [38] for *z*-scores on the TestMyBrain Visual Paired Associates Memory Test.

Given the rarity of CD and limited studies on this population, investigating specific domains can provide meaningful clinical information for application and further research exploration. Therefore, despite nonsignificant MANOVA findings, we conducted post hoc independent two-sample *t*-tests for each of the 12 domains that were included in the MANOVA models. No significant differences between the two groups emerged for any domain. For an example, see Figure 2, which demonstrates a two-group estimation plot of the visual memory domain.

Finally, in addition to *t*-tests, we also conducted chi-square tests of group differences in the proportion of individuals who performed greater than 1 standard deviation below the normative mean. The two groups only significantly differed on one measure of processing speed, where more people with NFA than people with CD performed at impaired levels. Average *z*-score performance on all cognitive tests was within normal limits (within one SD) for both groups (see Tables 2 and 3).

Self-reported history of depression revealed elevated symptoms of persistent depressive disorder (i.e., depressive symptomology for 2 years or more) for both groups (70% of people with CD; 35% of people with NFA; *X*^2^ = 4.91, *p* > 0.05), with 25% of people with NFA and 35% of people with CD reporting that their symptoms began prior to receiving their NFA/CD diagnosis. Further, 55% of both groups reported having been diagnosed with a depressive disorder in their lifetime. The two groups did not significantly differ in self-reported stress related to the COVID-19 pandemic (*t*(38) = −1.17, *p* > 0.05).

Across the three mediation models, the direct effect of group membership on cognitive outcome (memory, attention, and executive functioning) was not significant. The indirect effect of group membership on cognitive outcome via psychological functioning was also not significant (memory: IE = −0.035, 95%CI = −0.412, 0.360; attention: IE = 0.038, 95%CI = −0.562, 0.406; executive functioning: IE = 0.007, 95%CI = −0.164, 0.179). Given our interest in the relationship between psychological functioning and executive functioning, we also conducted bivariate correlations among the six cognitive domains and the six psychological domains, which revealed no significant correlations in the sample of individuals with CD.

In the group with CD, time since remission and processing speed were significantly and negatively correlated, such that longer periods of remission were associated with slower processing speed (see Table 4). This relationship was significant even after controlling for age and anxiety; however, when controlling for the presence of hypertension and DM, this relationship was no longer significant. No other variables were significantly correlated.

## 4. Discussion

The present study is one of the few to obtain an adequately sized sample of individuals with CD for hypothesis testing despite low incidence rates for CD (6.2–7.6 per million in the United States) [39], as well as an appropriately matched control group of individuals with NFA. The study was designed with the primary aim of comparing the cognitive and psychiatric profiles of individuals with biochemically remitted CD to those of people with NFAs. In addition, we tested the hypothesis that psychiatric functioning would partially mediate the relationship between group membership (CD vs. NFA) and cognitive functioning.

Findings were inconsistent with the hypothesis that individuals with CD in remission would display poorer performance on attention, executive functioning, visual–spatial functioning, memory, and processing speed tasks, relative to those individuals with NFAs. On average, both groups performed in the normal or above normal range across all domains, and, with only one exception, results revealed that the two groups did not significantly differ on neuropsychological measures. Specifically, levels of impairment across cognitive and psychiatric domains were statistically similar for the two groups on all tests except for one processing speed measure (Trails A errors; 0% CD; 20%NFA). Notably, however, there was substantive variability across individuals in both groups. Subtle decrements in performance were evident among at least a few members of each group in several domains, including attention (20%–25% CD; 15%–25% NFA), executive functioning (20%–30% CD; 5%–15% NFA), and visual memory (30% CD; 30% NFA).

These findings suggest the presence of persistent, but mild, neuropsychological difficulties for some individuals in both groups at an average of 4.9 years (4.8 for NFA patients; 5.0 for CD patients) postpituitary surgery. The absence of group differences in our study contrasts with findings from one other study that compared cognitive functioning between people with remitted CD and people with NFA [11]. One factor that may have led to these divergent findings is limitations in the computerized measures used to assess memory functioning in the present study. In particular, participants in the present study were administered memory tasks (i.e., visual/verbal paired associates) with delays of only 2.5–3 min. Further, previous research has suggested that impairments in memory among CD patients might be attributable to attention lapses [36]; in the absence of trial-by-trial data in the present study, we could not evaluate this possibility. It is also possible that the lack of significant differences represents a genuine phenomenon: publication bias, wherein negative results are underreported and underpublished in scientific journals (i.e., the “file drawer” effect).

Regarding psychiatric functioning, findings were consistent with the presence of substantive distress among many individuals with treated pituitary tumors, regardless of their secretion status. Self-reported positive affect was atypically low for half of the participants in both samples. Many participants in both groups also reported elevated negative affect, endorsing varied symptoms that included social anxiety (40% of people with CD; 35% of people with NFA) and somatization (30% of people with CD; 15% of people with NFA). Within both groups, 20% endorsed levels of anxiety above the clinical cutoff, which is higher than that for the general population (5%) [40]. Also, 15% of people with CD patients and 20% of people with NFA endorsed levels of depression above the clinical cutoff compared to only 9.1% of the general population [27]. The lack of clinically significant differences between groups may be due to inadequate power due to small sample sizes. It is also possible that rates of depression genuinely do not differ between the two patient populations. It merits note, however, that many participants in both groups endorsed minimal psychiatric symptomatology; the pattern of findings in the present study suggests that many individuals with pituitary tumors show emotional resilience after treatment.

Our findings regarding emotional functioning, similar to neuropsychological functioning, stand in contrast to a prior study [12]. Tiemensma et al. [12] found that people with remitted CD had higher scores than those with NFAs on measures of apathy, irritability, anxiety, negative affect, somatic arousal, and several personality scales. The average duration of remission for that study was 11 years, compared to 5 years in the present study. It is therefore possible that while individuals with NFA continue to recover over time, those with CD continue to experience psychiatric distress, such that data captured later in the course of recovery are more likely to show group differences. Notably, on a single self-report item regarding persistent depressive disorder symptoms, 70% of individuals with CD, relative to 35% of individuals with NFA, endorsed experiencing continuous depressive symptoms for at least 2 years. Half of the people with CD who reported enduring depression also noted that their symptoms began prior to receiving their CD diagnosis. This finding suggests that long-term depression symptoms might be more prevalent among people with CD and that they might benefit from appropriate treatment.

One potential reason why we failed to find significant cognitive or psychiatric differences between groups was an unexpectedly high prevalence of impairments among the NFA control group. Several studies have shown that patients with NFAs who have undergone surgery and/or radiotherapy perform significantly worse than healthy individuals following treatment on measures of memory [41, 42], executive functioning [41], processing speed [41], and attention [41]. The mechanism for these impairments is not clear; cognitive deficits could be due to hormonal disturbances, surgical complications, other prevalent comorbidities, or some combination of these. Additionally, the long-term course of neurocognitive functioning among people with NFA is not well understood; while some demonstrate reliable declines or stability in performance [41], several studies point to improvement over time [41, 42]. Therefore, the distribution of performance captured by our data could indicate a mixture of recovery profiles (i.e., stable, improvement, and decline) among individuals in the NFA sample.

Tests of the three models designed to examine if psychiatric functioning partially mediates the relationship between remitted CD and cognitive outcomes, specifically memory, executive functioning, and attention, did not yield significant results. Given the lack of significant differences between the two groups on almost every measure administered, it is not surprising that the relationships between group membership and cognitive outcomes (attention, memory, and executive functioning), as well as group membership and psychiatric functioning (composite score of depression and anxiety), also did not differ between groups. It is surprising, though, in light of the substantive evidence that anxiety and depression might impact or worsen cognitive functioning in other medical samples (e.g., TBI) [43], that these internalizing symptoms showed limited associations with cognitive outcomes in our study.

It may be that psychological and cognitive functioning are simply more weakly associated among individuals with CD and NFA than among individuals with other medical conditions (e.g., TBI and stroke). People with DM have also shown weak, nonsignificant associations between neurocognitive impairment and depression [44]. It is possible that pituitary tumors result in cognitive and psychiatric dysfunction through potentially independent mechanisms. People in remission from CD and NFA would also benefit from treatment of both neurocognitive and psychiatric dysfunction, as the treatment of one might not improve the other.

The present study was also inadequately powered to detect small or medium mediation effects. Even with the use of bias-corrected bootstrapping, we would require a sample size of 78 individuals or 462 individuals to detect medium or small mediation effect sizes, respectively [19]. Future longitudinal studies with larger samples of both groups are likely to better clarify the association between psychiatric and cognitive outcomes in people with remitted CD.

Bivariate correlations and chi-square tests between neuropsychological outcomes and several health and demographic factors revealed one valid significant association. Time since remission was negatively correlated with processing speed, even after controlling for age and anxiety, such that the more time that had passed since achieving remission, the slower the processing speed. It is not clear why CD, and/or its treatment, might be associated with a progressive decline in processing speed over time. However, given persistently widespread reductions of white matter integrity in people with long-term remitted CD [15, 45] and the positive association between white matter integrity and processing speed in people with remitted CD [15] and healthy individuals [46], it is possible that white matter health continues to decline among people with CD, resulting in worsening processing speed over time. Further, several common comorbidities such as hypertension and DM, which often also persist after treatment of CD [22], might confer increased risk for white matter lesions [47] and, consequently, slower processing speed [48]. Indeed, in the present sample, more people with CD than those with NFA had hypertension and/or DM at examination. Exploratory analyses revealed that the relationship between processing speed and time since CD remission was no longer significant after controlling for the presence of hypertension and DM. This underlines the importance of evaluation and treatment of persistent comorbidities in patients with CD in remission. Future CD studies that incorporate the use of neuroimaging and larger sample sizes would be especially helpful in confirming or disproving this potential mechanism.

The present study was conducted during the COVID-19 pandemic. As a result, the findings may have been influenced by participants' psychological and physiological responses to the virus itself and/or the pandemic. Our sample endorsed similar levels of COVID-related stress as a healthy sample [49]; however, according to the American Psychological Association, average reported stress levels among US adults were higher in January 2021 than at the beginning of the pandemic in April 2020, suggesting that many healthy Americans continued to experience emotions associated with prolonged stressful events. Although stress associated with the pandemic and other societal events of 2020 and 2021 (e.g., racial injustice and breach of the US Capitol) is difficult to disentangle from anxiety and mood symptoms typically associated with remitted CD, it is important to consider the potential compounding effects of these stressors on underlying psychological distress associated with having and undergoing treatment for a pituitary tumor. The similar rates of anxiety, depression, low positive affect, and somatization reported by the individuals with CD and individuals in the NFA control group might reflect the impact of shared contextual stressors; these factors could have overshadowed any prolonged effects of cortisol produced by a CD tumor. In future studies, two potential ways to disentangle stress associated with COVID-19 from the effects of a pituitary tumor include (1) comparing neuropsychological performance among patients with CD to that of a healthy, matched sample and (2) repeating neuropsychological testing after the COVID-19 pandemic has ended.

Another limitation of the present study was the reliance on convenience sampling from patients at a single academic medical center. This approach may have limited generalization of findings to the broader patient population, many of whom do not have access to surgical interventions at tertiary referral centers. In fact, in a study of a nationwide sample of patients who underwent TSS for CD between 2002 and 2010, patients with Medicare and Medicaid insurance were at a disadvantage to patients with private insurance [50]. Our use of convenience sampling also resulted in the overrepresentation of White patients in the present sample, further decreasing the generalizability of findings.

Finally, while digital neuropsychology offers several advantages, including improved accessibility, precise behavior measurement (e.g., reaction time in milliseconds), broad standardization, feasibility, and low cost, it also has disadvantages. First, the TMB toolkit was designed to be self-administered without technician involvement. This precludes oversight of participant task engagement and collection of qualitative data regarding the patient's approach to tests. The toolkit also has psychometric shortcomings. Only five of the eight tests used in the present study (Digit Symbol Matching, Matrix Reasoning, Digit Span, Verbal Paired Associates, and Gradual Onset CPT) have been validated against in-person assessments, and only three (Digit Symbol Matching, Matrix Reasoning, and Digit Span) have been validated against clinical neuropsychological assessments. Further, several of the tests have yet to be validated in clinical or medical samples. Although the TMB battery is currently being used in large-scale research studies of psychopathology and medical disorders (e.g., NIMH Aurora study, Harvard Football Players Health Study, High School and Beyond, and 23andme), few study results are yet available, forcing us to exercise caution in interpreting the results of the present study, whose participants comprised entirely medical patients.

Despite these limitations, the present study has several strengths. First, a strength of the present study is the assessment of psychiatric factors in conjunction with cognitive functioning. Evaluating these areas in parallel not only yielded a holistic cognitive and psychiatric profile of CS patients in remission but also allowed us to examine whether there was a significant interplay between psychiatric and cognitive functioning in this population. Second, data were collected using well-validated and reliable digital neuropsychological measures of several cognitive domains. Most existing studies of cognitive functioning in people with remitted CD have focused on only a few cognitive domains, typically memory and executive functioning [9, 11]. Further, because we compared functioning among individuals with CD to that of members of an appropriate comparison group (individuals with NFAs), we were well equipped to detect a profile that was specific to the effects of chronic hypercortisolism, were it to have been present. Finally, the present study contributes an important finding to the literature—both groups share subtle decrements in neuropsychological functioning. It will be important for future research to explore factors that increase the risk for long-term neuropsychological dysfunction among some, but not all, people with CD and NFA.

With regard to study design, future studies should recruit larger samples if possible. Further, random sampling of participants from varied geographical locations, treatment hospitals (e.g., academic and nonacademic), racial and ethnic backgrounds, and socioeconomic statuses (e.g., insurance access) is important for increasing the generalizability of findings and adequate recommendations for all tumor patients. Given the challenges inherent in recruiting large, representative samples from relatively rare patient populations, there would also be value in research that takes an idiographic approach that centers on in-depth examination of individual patients who represent varied demographic and clinical subgroups. Such work would allow for the generation of data about patients to whom it may be difficult to generalize findings from nomothetic studies. Additionally, prospective, longitudinal studies are critical to establishing causality, delineating recovery trajectories (e.g., stability, decline, and improvement), and exploring protective and risk factors for various outcomes.

## Figures and Tables

**Table 1:** Included participant characteristics for individuals with CD and NFA.

	**Cushing's disease (** **n** ** = 20)**	**Nonfunctioning adenoma (** **n** ** = 20)**	**Chi-square value/** **t** **-score**
Gender			*X* ^2^ = 0.00
Female (%)	80%	80%	
Male (%)	20%	20%	
Race			*X* ^2^ = 4.00
American Indian (%)	5%	0%	
Black/African American (%)	15%	30%	
Latinx (%)	5%	0%	
Multiracial (%)	5%	0%	
White (%)	70%	70%	
Age at examination (SD)	45.25 (11.51)	48.05 (11.69)	*t* = 0.763
Age at diagnosis (SD)	39.5 (11.03)	42.2 (11.143)	*t* = 0.770
Years of education (SD)	14.95 (2.74)	15.3 (1.92)	*t* = −0.467
Months since remission^a^ (SD)	61.6 (44.13)	—	*—*
Time between most recent surgery and current examination	5.04 (4.10)	4.82 (3.23)	*t* = 0.191
History of pituitary radiotherapy	15%	15%	*X* ^2^ = 0.00
Characteristics present at examination			
Receiving hydrocortisone replacement	20%	15%	*X* ^2^ = 0.17
Hypertension	50%	5%	*X* ^2^ = 10.16^∗∗^
Diabetes mellitus (DM)	50%	10%	*X* ^2^ = 7.62^∗^
Hypogonadism	10%	0%	*X* ^2^ = 2.11
Hypothyroidism	40%	20%	*X* ^2^ = 1.90

^a^Remission only applies to the group with CD and indicates resolution of hypercortisolism.

∗*p* < 0.05.

∗∗*p* < 0.001.

**Table 2:** Comparison of frequency of impaired performance on cognitive tests between individuals with CD and NFA.

**Measure**	**Chi-square value**	**p** ** value**	**% impaired nonfunctioning** **adenoma (** **n** **), avg ** **z** ** -score**	**% impaired Cushing's disease** **(** **n** **), avg ** **z** ** -score**
Sustained attention				
CPT impulsivity	0.625	0.429	15% (3), *z* = 0.02	25% (5), *z* = 0.16
CPT detectability	0.143	0.705	25% (5), *z* = −0.30	20% (4), *z* = −0.19
Processing speed and executive functioning				
Trails A total time	2.057	0.151	20% (4), *z* = 0.05	5% (1), *z* = 0.44
Trails A errors	4.444	0.035⁣^∗^	20% (4), *z* = −0.12	0% (0), *z* = 0.44
Trails B total time	2.057	0.151	5% (1), *z* = 0.13	20% (4), *z* = −0.21
Trails B errors	0.000	1.000	5% (1), *z* = 0.19	5% (1), *z* = 0.05
CRT	0.625	0.429	15% (3), *z* = −0.34	25% (5), *z* = −0.37
CRT accuracy	1.026	0.311	0% (0), *z* = 0.29	5% (1), *z* = 0.12
Visual learning and memory				
Visual memory	0.000	1.000	30% (6), *z* = −0.05	30% (6), *z* = −0.23
Verbal learning and memory				
Verbal memory	0.000	1.000	20% (2), *z* = 0.03	10% (2), *z* = 0.16
Visual abstract reasoning				
Matrix reasoning	1.111	0.292	5% (1), *z* = 0.27	15% (3), *z* = −0.22
Auditory attention and working memory				
Forward digit span	2.057	0.151	5% (1), *z* = 0.36	20% (4), *z* = −0.16
Backward digit span	1.290	0.256	15% (3), *z* = −0.08	30% (6), *z* = −0.14
Processing speed				
Digit symbol matching	0.125	0.723	25% (5), *z* = −0.49	30% (6), *z* = −0.54

*Note:* Impairment was characterized by performance greater than 1 standard deviation below the normative mean.

Abbreviations: CPT = continuous performance test, CRT = choice reaction time.

⁣^∗^*p* < 0.05.

⁣^∗∗^*p* < 0.01.

⁣^∗∗∗^*p* < 0.001.

**Table 3:** Comparison of frequency of impaired performance on psychiatric measures between CD and NFA patients.

**Measure**	**Chi-square value**	**p** ** value**	**% impaired NFA (** **n** **), avg score**	**% impaired CD (** **n** **), avg score**
Positive affect	0.000	1.000	50% (10), *M* = 27.63	50% (10), *M* = 26.15
Social anxiety	0.107	0.744	35% (7), *M* = 21.85	40% (8), *M* = 21.60
Somatization	1.290	0.256	15% (3), *M* = 6.45	30% (6), *M* = 8.45
Depression	0.173	0.677	20% (4), *M* = 5.75	15% (3), *M* = 6.65
Anxiety	0.000	1.000	20% (4), *M* = 6.46	20% (4), *M* = 6.15

**Table 4:** Bivariate correlations in the group with CD between cognitive/psychological domains and measures of illness duration.

	**Age at diagnosis**	**Duration of hypercortisolism**	**Time since remission**
Attention	0.249	−0.035	−0.228
Processing speed	0.411	−0.002	−0.478⁣^∗^
Executive functioning	0.232	0.038	−0.129
Visual–spatial skills	−0.238	0.029	−0.134
Visual memory	−0.238	0.029	−0.134
Verbal memory	0.289	0.005	−0.389
Anxiety	0.140	−0.118	−0.412
Depression	−0.050	0.015	−0.398
Health and disability	0.311	−0.287	−0.138
Positive affect	0.271	0.094	0.043
Somatic concerns	0.040	−0.258	0.237
Social anxiety	−0.251	−0.207	0.253

*Note:* All cognitive domains were represented by z-scores, while psychiatric domains were represented by total scores on their respective measures.

⁣^∗^*p* < 0.05.

**Table 5:** Computerized neuropsychological testing by domain.

**Domain**	**Test(s)**
Effort testing	- Reliable Digit Span

Attention and working memory	- TMB Digit Span
	- TMB Gradual Onset Continuous Performance Test

Processing speed	- TMB Digit Symbol Matching
	- TMB Trail-Making Test Part A

Visual/spatial skills	- TMB Matrix Reasoning

Verbal memory	- TMB Verbal Paired Associates Memory

Visual memory	- TMB Visual Paired Associates Memory

Executive functioning	- TMB Trail-Making Test Part B - TMB Choice Reaction Time

Psychological functioning	- Positive and Negative Affect Schedule (PANAS)
	- Brief Fear of Negative Evaluation (BFNE)
	- Patient Health Questionnaire-15 item (PHQ-15; Somatic Symptom scale)
	- TMB World Health Organization Disability Assessment Schedule-2.0 (WHODAS 2.0)
	- TMB Patient Health Questionnaire-8 item (PHQ-8; Depression scale)
	- TMB Generalized Anxiety Disorder Questionnaire-7 item (GAD-7)

Abbreviation: TBM toolkit, TestMyBrain Digital Neuropsychology Toolkit.

**Table 6:** Psychometric properties of included measures.

**Measure**	**Internal consistency (** **α** **)**	**Validity**
TMB Digit Span	—	—
TMB Gradual Onset Continuous Performance Test	0.78	High
TMB Digit Symbol Matching	0.93	High
TMB Trail-Making Test Part A & B	0.97	High
TMB Matrix Reasoning	0.89	High
TMB Visual Paired Associates Memory	0.79	Med
TMB Verbal Paired Associates Memory	0.82	Med
TMB Choice Reaction Time	0.95	Med
TMB PHQ-8	0.86	High
TMB GAD-7	0.92	High
TMB WHODAS-2.0	0.98	High
BFNE	0.966^a^	High
PANAS-PA	0.925^a^	High
PHQ-15	0.804^a^	High

*Note:* Unless otherwise noted, all TMB Cognitive test psychometric properties were taken from the TestMyBrain NIMH Research Domain Criteria (RDOC) field test battery report [51]. Psychometric sources for the remaining tests are as follows: TMB Trail Making Test A & B [52]; PHQ-8 [27]; GAD-7 [26]; WHODAS-2.0 [28]; BFNE [30]; PANAS-PA [29]; PHQ-15 [53].

Abbreviations: BFNE = Brief Fear of Negative Evaluation scale, GAD-7 = Generalized Anxiety Disorder scale, PANAS-PA = Positive and Negative Affect Schedule—Positive Affect Scale, PHQ-8 = Patient Health Questionnaire Depression scale, PHQ-15 = Patient Health Questionnaire Somatic Symptom scale, TMB = TestMyBrain, WHODAS-2.0 = World Health Organization Disability Assessment Schedule.

^a^Internal consistency of item responses endorsed by the present study sample.

## Data Availability

The dataset supporting this study is not freely available given participants' privacy protection.

## Appendix A: Computerized Testing Measures

All testing was conducted remotely via computer, using online-accessible neuropsychological tests and questionnaires that patients were able to complete in their own homes. Cognitive functioning was assessed using the TMB Digital Neuropsychology Toolkit. Measures are described below, and additional details can also be found at http://testmybrain.org.

### Measures

• TMB Digit Symbol Matching: The Digit Symbol Matching test is a measure of processing speed. Participants are shown symbol–number keys on the screen and asked to match as many symbols and numbers as possible in 90 s.
• TMB Matrix Reasoning: This test measures visual and spatial skills. Participants are asked to identify the image that best completes the pattern in a series.
• TMB Digit Span: This test is a measure of attention and working memory. Participants are asked to recall sequences of digits of increasing length in the same (forward digit span) or opposite (backward digit span) order as presented.
• TMB Trail-Making Test: This test measures processing speed (Part A) and executive functioning (Part B) skills. In Part A of the test, participants are asked to connect a series of numbers in order. In Part B of the test, participants are asked to connect numbers and letters in alternate ascending order.
• TMB Choice Reaction Time: This is a test of processing speed and executive functioning. The participant is asked to select the direction of the arrow that is in a different color from the other arrows.
• TMB Verbal Paired Associates Memory: The test measures verbal memory and attention. Participants learn a set of word pairs and are then asked to recognize the accurate match for a given word following a 2–3 min delay and interference.
• TMB Visual Paired Associates Memory: This test measures visual memory and attention. Participants learn a set of picture (sceneries) pairs and are then asked to recognize the accurate match for a given picture following a 2–3 min delay and interference.
• TMB Gradual Onset Continuous Performance Test: This is a measure of sustained attention and response inhibition. Images rapidly transition from mountain images to city images, and the participant is asked to press a key when a city image appears and to not press any key when a mountain image appears. Mountain images appear 10%–20% of the time.

The psychometric properties of study measures are included in the following table.

## References

[1] Fleseriu M., Auchus R., Bancos I. (2021). Consensus on diagnosis and management of Cushing's disease: a guideline update. *The Lancet Diabetes & Endocrinology*.

[2] Pereira A. M., Tiemensma J., Romijn J. A., Biermasz N. R. (2012). Cognitive impairment and psychopathology in patients with pituitary diseases. *The Netherlands Journal of Medicine*.

[3] Dorn L. D., Burgess E. S., Dubbert B. (1995). Psychopathology in patients with endogenous Cushing's syndrome: ‘atypical’ or melancholic features. *Clinical Endocrinology*.

[4] Sonino N., Fava G. A., Raffi A. R., Boscaro M., Fallo F. (1998). Clinical correlates of major depression in Cushing’s disease. *Psychopathology*.

[5] Starkman M. N. (2013). Neuropsychiatric findings in Cushing syndrome and exogenous glucocorticoid administration. *Endocrinology and Metabolism Clinics*.

[6] Haskett R. F. (1985). Diagnostic categorization of psychiatric disturbance in Cushing's syndrome. *The American Journal of Psychiatry*.

[7] Dorn L. D., Burgess E. S., Friedman T. C., Dubbert B., Gold P. W., Chrousos G. P. (1997). The longitudinal course of psychopathology in Cushing’s syndrome after correction of hypercortisolism. *The Journal of Clinical Endocrinology & Metabolism*.

[8] Sonino N., Fava G. A. (2001). Psychiatric disorders associated with Cushing’s syndrome. *CNS Drugs*.

[9] Alsumali A., Cote D. J., Regestein Q. R. (2017). The impact of transsphenoidal surgery on neurocognitive function: a systematic review. *Journal of Clinical Neuroscience*.

[10] Forget H., Lacroix A., Bourdeau I., Cohen H. (2016). Long-term cognitive effects of glucocorticoid excess in Cushing’s syndrome. *Psychoneuroendocrinology*.

[11] Tiemensma J., Kokshoorn N. E., Biermasz N. R. (2010). Subtle cognitive impairments in patients with long-term cure of Cushing’s disease. *The Journal of Clinical Endocrinology & Metabolism*.

[12] Tiemensma J., Biermasz N. R., Middelkoop H. A. M., van der Mast R. C., Romijn J. A., Pereira A. M. (2010). Increased prevalence of psychopathology and maladaptive personality traits after long-term cure of Cushing’s disease. *The Journal of Clinical Endocrinology & Metabolism*.

[13] Cavagnini F., Scacchi M., Pecori F. G. (2008). Hypopituitarism in Cushing's disease. *Journal of Endocrinological Investigation*.

[14] Starkman M. N., Schteingart D. E. (1981). Neuropsychiatric manifestations of patients with Cushing's syndrome: relationship to cortisol and adrenocorticotropic hormone levels. *Archives of Internal Medicine*.

[15] Pires P., Santos A., Vives-Gilabert Y. (2017). White matter involvement on DTI-MRI in Cushing’s syndrome relates to mood disturbances and processing speed: a case-control study. *Pituitary*.

[16] Stomby A., Salami A., Dahlqvist P. (2019). Elevated resting-state connectivity in the medial temporal lobe and the prefrontal cortex among patients with Cushing’s syndrome in remission. *European Journal of Endocrinology*.

[17] Cohen J. (1988). *Statistical Power Analysis for the Behavioral Sciences (2. Auflage)*.

[18] Erdfelder E., Faul F., Buchner A. (1996). GPOWER: a general power analysis program. *Behavior Research Methods, Instruments, & Computers*.

[19] Fritz M. S., MacKinnon D. P. (2007). Required sample size to detect the mediated effect. *Psychological Science*.

[20] Goswami N., Handa T., Pappy A., Veledar E., Oyesiku N., Ioachimescu A. (2019). SUN-LB074 racial distribution, presentation and outcome in acromegaly and Cushing's disease: a tertiary referral center study in 220 patients. *Journal of the Endocrine Society*.

[21] Yaneva M., Kalinov K., Zacharieva S. (2013). Mortality in Cushing’s syndrome: data from 386 patients from a single tertiary referral center. *European Journal of Endocrinology*.

[22] Schernthaner-Reiter M. H., Siess C., Gessl A. (2019). Factors predicting long-term comorbidities in patients with Cushing’s syndrome in remission. *Endocrine*.

[23] Wechsler D. (2008). *Wechsler Adult Intelligence Scale—Fourth Edition*.

[24] Chaytor N. S., Barbosa-Leiker C., Germine L. T., Fonseca L. M., McPherson S. M., Tuttle K. R. (2021). Construct validity, ecological validity and acceptance of self-administered online neuropsychological assessment in adults. *The Clinical Neuropsychologist*.

[25] Germine L., Nakayama K., Duchaine B. C., Chabris C. F., Chatterjee G., Wilmer J. B. (2012). Is the web as good as the lab? Comparable performance from web and lab in cognitive/perceptual experiments. *Psychonomic Bulletin & Review*.

[26] Spitzer R. L., Kroenke K., Williams J. B. W., Löwe B. (2006). A brief measure for assessing generalized anxiety disorder: the GAD-7. *Archives of Internal Medicine*.

[27] Kroenke K., Strine T. W., Spitzer R. L., Williams J. B. W., Berry J. T., Mokdad A. H. (2009). The PHQ-8 as a measure of current depression in the general population. *Journal of Affective Disorders*.

[28] Üstün T. B., Kostanjsek N., Chatterji S., Rehm J. (2010). *Measuring Health and Disability: Manual for WHO Disability Assessment Schedule WHODAS 2.0*.

[29] Watson D., Clark L. A., Tellegen A. (1988). Development and validation of brief measures of positive and negative affect: the PANAS scales. *Journal of Personality and Social Psychology*.

[30] Carleton R. N., Collimore K. C., McCabe R. E., Antony M. M. (2011). Addressing revisions to the brief fear of negative evaluation scale: measuring fear of negative evaluation across anxiety and mood disorders. *Journal of Anxiety Disorders*.

[31] Kroenke K., Spitzer R. L., Williams J. B. W., Löwe B. (2010). The patient health questionnaire somatic, anxiety, and depressive symptom scales: a systematic review. *General Hospital Psychiatry*.

[32] Blake D. D., Weathers F. W., Nagy L. M. (1995). The development of a clinician-administered PTSD scale. *Journal of Traumatic Stress*.

[33] Myers A., Rosen J. C. (1999). Obesity stigmatization and coping: relation to mental health symptoms, body image, and self-esteem. *International Journal of Obesity*.

[34] Taylor S., Landry C. A., Paluszek M. M., Fergus T. A., McKay D., Asmundson G. J. G. (2020). Development and initial validation of the COVID stress scales. *Journal of Anxiety Disorders*.

[35] McLean C. P., Cloitre M. (2020). Coronavirus stressor survey.

[36] Na S., Fernandes M. A., Ioachimescu A. G., Penna S. (2020). Neuropsychological and emotional functioning in patients with Cushing’s syndrome. *Behavioural Neurology*.

[37] Hayes A. F. (2017). *Introduction to Mediation, Moderation, and Conditional Process Analysis: A Regression-Based Approach*.

[38] Ho J., Tumkaya T., Aryal S., Choi H., Claridge-Chang A. (2019). Moving beyond *P* values: data analysis with estimation graphics. *Nature Methods*.

[39] Broder M. S., Neary M. P., Chang E., Cherepanov D., Ludlam W. H. (2015). Incidence of Cushing's syndrome and Cushing's disease in commercially-insured patients <65 years old in the United States. *Pituitary*.

[40] Löwe B., Decker O., Müller S. (2008). Validation and standardization of the generalized anxiety disorder screener (GAD-7) in the general population. *Medical Care*.

[41] Butterbrod E., Gehring K., Voormolen E. H. (2020). Cognitive functioning in patients with nonfunctioning pituitary adenoma before and after endoscopic endonasal transsphenoidal surgery. *Journal of Neurosurgery*.

[42] Pertichetti M., Serioli S., Belotti F. (2020). Pituitary adenomas and neuropsychological status: a systematic literature review. *Neurosurgical Review*.

[43] Barker-Collo S., Jones K., Theadom A. (2015). Neuropsychological outcome and its correlates in the first year after adult mild traumatic brain injury: a population-based New Zealand study. *Brain Injury*.

[44] Solanki R. K., Dubey V., Munshi D. (2009). Neurocognitive impairment and comorbid depression in patients of diabetes mellitus. *International Journal of Diabetes in Developing Countries*.

[45] Pires P., Santos A., Vives-Gilabert Y. (2015). White matter alterations in the brains of patients with active, remitted, and cured Cushing syndrome: a DTI study. *American Journal of Neuroradiology*.

[46] Magistro D., Takeuchi H., Nejad K. K. (2015). The relationship between processing speed and regional white matter volume in healthy young people. *PLoS One*.

[47] Wang D. Q., Wang L., Wei M. M. (2020). Relationship between type 2 diabetes and white matter hyperintensity: a systematic review. *Frontiers in Endocrinology*.

[48] Ozaldemir I., Iyigun G., Malkoc M. (2020). Comparison of processing speed, balance, mobility and fear of falling between hypertensive and normotensive individuals. *Brazilian Journal of Physical Therapy*.

[49] Asmundson G., Taylor S. (2020). Coronaphobia revisted: a state-of-the-art on pandemic-related fear, anxiety, and stress. *Journal of Anxiety Disorders*.

[50] Wilson D., Jin D. L., Wen T. (2015). Demographic factors, outcomes, and patient access to transsphenoidal surgery for Cushing's disease: analysis of the nationwide inpatient sample from 2002 to 2010. *Neurosurgical Focus*.

[51] Passell E., Dillon D. G., Baker J. T. (2019). *Digital cognitive assessment: Results from the TestMyBrain NIMH Research Domain Criteria (RDoC) field test battery report*.

[52] Singh S., Strong R. W., Jung L. (2021). The TestMyBrain Digital Neuropsychology Toolkit: Development and Psychometric Characteristics. *Journal of Clinical and Experimental Neuropsychology*.

[53] Kroenke K., Spitzer R. L., Williams J. B. (2002). The PHQ-15: validity of a new measure for evaluating the severity of somatic symptoms. *Psychosomatic Medicine*.

